# Does It Look Good or Evil? Children’s Recognition of Moral Identities in Illustrations of Characters in Stories

**DOI:** 10.3389/fpsyg.2021.552387

**Published:** 2021-04-23

**Authors:** Núria Obiols-Suari, Josep Marco-Pallarés

**Affiliations:** ^1^Department of Theory and History of Education, Group Research of Moral Education GREM, Institute of Educational Research IRE, University of Barcelona, Barcelona, Spain; ^2^Department of Cognition, Development and Educational Psychology, Institute of Neurosciences, University of Barcelona, Barcelona, Spain; ^3^Cognition and Brain Plasticity Group, Bellvitge Biomedical Research Institute (IDIBELL), L’Hospitalet de Llobregat, Spain

**Keywords:** moral judgements, moral intuitions, moral identity, children, skin conductance

## Abstract

Children usually use the external and physical features of characters in movies or stories as a means of categorizing them quickly as being either good or bad/evil. This categorization is probably done by means of heuristics and previous experience. However, the study of this fast processing is difficult in children. In this paper, we propose a new experimental paradigm to determine how these decisions are made. We used illustrations of characters in folk tales, whose visual representations contained features that were compatible or incompatible with the moral identity of the characters. Sixteen children between 8 and 10 years old participated in the experiment. We measured their electrodermal activity when they were listening to the story and looking at pictures of the characters. Results revealed a higher increase in skin conductance when the illustrations showed a moral condition that was incompatible with the actions of a character than when they showed one that was compatible. These results suggest that children make fast decisions about the moral identity of characters based on their physical features. They open up new possibilities in the study of the processing of moral decisions in children.

## Introduction

A key skill in social interactions is to determine who deserves to be trusted and who does not. People usually rely on their previous experience with others and especially on their history with the person to be trusted. However, when fast decisions have to be taken (e.g., when a stranger approaches us in the street and ask us a question), they are based on heuristics, that is, simple rules that allow us to make a fast choice even at risk of failing. Interestingly, these non-reflective decisions are used not only in contexts in which rapid decisions are needed, but also in situations in which time is not a key factor. Different theoretical accounts have argued that most moral judgements are made almost automatically (Haidt, 2001). Therefore, the social intuitionist model proposes that moral judgement is closer to an emotion, or emotional intuition, than to reasoned, rational processing and that such judgements appear suddenly and effortlessly based on feelings and intuitions, while rational arguments are just *post hoc* justifications (Greene and Haidt, 2002). On the other hand, rationalist models have emphasised how people develop self-moral identity and moral schemas. Under this perspective, moral judgement is a moral reasoning, and the relationship between moral judgement and moral action (Kohlberg and Candee, 1984) is complex and influenced by many variables such as educational opportunities (Stephens, 2018).

Either rationally or intuitively, both adults and children are able to quickly categorise people – as well as characters in movies and illustrations – as being either “good” or “evil” by using different features. For example, previous studies have identified that some physical traits play an important role in such decisions and when additional information is not available, both children and adults rate unattractive characters as evil and attractive as good in moral terms (Griffin and Langlois, 2006), although attractiveness is not unambiguously associated with morality. Other important physical attributes used in moral judgements are facial features, especially the eyebrows or mouth (Dotsch and Todorov, 2012). Certainly, in children’s literature, it is more common to find ugly-bad and beautiful-good characters than the opposite. Interestingly, fMRI studies have found that there are shared neural mechanisms for aesthetic and moral judgements (Tsukiura and Cabeza, 2010). In addition, other physical characteristics, especially those reflecting some kind of threat (e.g., animals with claws, open mouths, sharp teeth, or convex eyes, Lee and Kang, 2012) might be used in illustrations to induce moral identities.

The consistency of all these previous results notwithstanding, very few studies have been devoted to determining the degree of robustness of such perceptions, and how quickly and reliably children are able to change the first automatic moral attitudes associated with a certain character on the basis of their actions. The objective of the present paper was to determine whether children automatically detected incongruences between the moral identity of a character and the moral identity induced by his/her physical appearance, and whether children were able to change it based on the character’s actions.

In order to reach this goal, we designed a new experimental paradigm to study the moral identities perceived by children, as well as how they change, by using the visual expression of characters in folk tales. Folk tales were selected ahead of other narrative texts, such as novels or contemporary stories, because of the familiarity children have with their contents. In addition, folk tales offer a rich environment in which to test the moral identities of characters, as their decisions often divide them along the lines of being either morally good or evil. Finally, folk tales present an optimal narrative construction that makes them very suitable for use in an experimental context. When they originated, these tales were transmitted orally. Later, they were compiled in writing (see, e.g., the well-known examples of Charles Perrault, Wilheim and Jakob Grimm, or Aleksandr Nikoláyevich Afanásiev). Folk tales (AKA fables and fairy tales) not only contain warnings (e.g., do not walk alone into the woods) but also show good-desirable (e.g., listening to old people) or bad-regrettable (e.g., betraying, abusing power, and stealing) behaviours. These actions are performed by good, evil, or ambiguous characters who have to face different situations, very often of a moral nature. Interestingly, people are able to identify these behaviours and associate them with the characters.

In the experiment, children were required to evaluate the moral identity of some characters from illustrations, both before and after listening to the folk tale. Children’s literature commonly presents a wide repertoire of illustrations, which (like images in general) have historically been used to transmit a multiplicity of messages. This has led to many studies about their analysis (Nodelman, 1988; Doonan, 1993; Wagner, 2013); about the variety of interactions between text and images (Nikolajeva and Scott, 2001), and their contribution to textual comprehension (Brookshire et al., 2002; Baird et al., 2016). In fact, illustrations explain and expand information about the characters of the plot. One of the most valuable pieces of information provided by such illustrations is about the moral identity of the characters. In our experiment, some illustrations coincided with their perceived moral identities of the characters of the tale, while others did not. We hypothesised that children would be able to identify the illustrations of different characters as good or evil on the basis of some physical characteristics and traits. In order to have an objective measure of the autonomic response system to incongruent illustrations, we used electrodermal activity. This measure reflects autonomic changes in the skin’s electrical conductance due to sweat secretion and is associated with sympathetic response. Skin Conductance Response (SCR) is a biomarker of arousal and it has been studied in a wide variety of studies. Increases in SCR have consistently been found to be associated with salient stimuli, for example, in response to sounds which have been previously paired with harmful stimuli (Dawson and Schell, 1982), or in the anticipation (Agren et al., 2019) and presentation of monetary gains or losses (Mas-Herrero et al., 2014; Agren et al., 2019). SCR also increases with the impact of the content of emotional images (Lang et al., 1993) and with the intensity of the enjoyment when people listen to pleasant music (Mas-Herrero et al., 2014). Interestingly, different studies have also found that SCR is sensitive to incongruent or conflict events. For example, Mitchell (2006) found increased SCR for incongruent conditions compared to congruent conditions both in a Stroop task and an auditory emotional interference task. Similarly, Zhang et al. (2012) found increased SCR in a stop signal task in high conflict trials compared to low conflict ones. Therefore, based on these studies, we hypothesised that the presentation of incompatibility between *a priori* and real moral identity (that is, the one derived from the actions of the character during the course of the story) would induce an increase in the autonomous nervous system response for these characters compared to the compatible ones while listening to the tale, being reflected by an increase in the SCR. Finally, we hypothesised that the perception of moral identity of those illustrations incompatible with the behaviour of the characters during the story would change after listening to the tale.

## Materials and Methods

### Participants

Sixteen children (age = 8.9 ± 0.8 years old, range 8–10 years old, five girls) participated in the experiment. The experiment was approved by the ethics committee of the University of Barcelona and followed the indications of the Helsinki declaration. The participation was voluntarily, and all parents signed an informed consent before it.

### Experimental Design

We used a classical children’s folk tale, The Treasure of the Blue Mountain (in Catalan, El Tresor de la Muntanya Blava). The main plot narrates the adventures of a boy who has to find the treasure of the blue mountain in order to inherit the kingdom of his father after his two older brothers have failed in the mission. The main character goes through different difficulties and is helped by people he meets along his journey. He finds the treasure and rescues a princess, but is betrayed by his two brothers, who try to kill him. Nevertheless, the prince finally reaches his goals, inherits the kingdom, and marries the princess. This story was compiled and transcribed by a writer of folk tales at the beginning of the 20th century (Serra i Boldú, 1922) and is particularly rich in terms of its characters, who present different motivations and moral decisions, making their moral identity easily recognisable. The story has a classical structure and many of the characters and prototypical functions of folk tales (Propp, 1968). It is also long enough to allow sufficient variety of characters. In addition, it is not very well known, which meant that it was similarly new to almost all participants (only one participant knew the tale beforehand). A professional storyteller recorded the tale in Catalan. Its duration was 23 min and 18 s (3,539 words). In order to check our assumptions about the moral identity of the characters of the tale, a different group of 10 children (six girls, 8 ± 0.9 years old) listened to the tale without images and rated the characters in a 0 (very bad) to 1 (very good) visual analog scale on the basis of their behaviour in the story. Children consistently rated the good characters with higher scores (0.86 ± 0.05) than bad characters (0.20 ± 0.13, W = 0, *p* = 0.002). These results clearly show that children correctly recognised the moral identity of the actions of the characters.

The day before the experiment, children listened to the tale at home in order to be sure that they knew the actions and moral identity of the characters. Then, on the day of the experiment, the children listened again to the story, but at certain moments, when a character had a remarkable role, the image of the character appeared on the screen for 10 s. Three images were compatible with the moral attitude of their character and three were not compatible (see Table 1). In addition, two images presenting characters with neutral role in the tale were also presented as a control condition. Children were instructed to pay attention to the story and to the images appearing on the screen corresponding to the characters.

**Table 1:** Illustrations presented and their corresponding moral congruency with the content of the tale.

	Moral identity (based on the actions in the tale)	Moral identity (based on the ratings of the image)	Condition	Number of times of image presentation
Little brother (main character)	Good	Good	Congruent	3
Second brother	Bad	Bad	Congruent	3
Giant	Bad	Bad	Congruent	1
Eldest brother	Bad	Good	Incongruent	3
Princess	Good	Bad	Incongruent	3
Old lady	Good	Bad	Incongruent	3
King	Neutral	Neutral	-	3
Servant	Neutral	Neutral	-	1

Before and after listening to the tale, participants rated 30 images presented in randomized order: Twenty-two of the images did not appear in the story and eight did. Each image was displayed on the screen and participants had to rate on a visual analog scale, presented on the screen, how good or bad the characters were from 0 (extremely good) to 1 (extremely bad). If children asked what it meant to be good or bad, they were told to decide on the basis of the image how they thought the characters were. Due to an error in the programming of the task, one of the neutral images presented in the tale (the king) was not presented in the evaluation but this did not affect posterior analysis.

The 30 sample pictures were extracted from illustrations in books of children’s tales, except for two that were drawn by the first author of this paper. Pictures were selected to cover a wide range of moral identities, from good to evil, with some of them presenting a neutral identity (12 *a priori* good, 12 evil, and six neutral). Moreover, each group had the same number of women and men. The reason to include the neutral characters was to determine whether children would be able to rate medium moral identities and not just extremes. We also changed the pictures to remove some elements obviously related to wickedness and goodness. For example, those elements that clearly pointed to an evil character, such as skulls on clothes or witches’ hats, were removed from the illustration. Other elements were also deleted when they were clearly linked to a very popular tale (e.g., an apple in the hand of an old lady could recall the stepmother of Snow White). In addition, none of characters was performing any action, in order to avoid interference with the decisions. Finally, all characters were in a similar position in a medium plane; they were from different tales and drawn by different artists to reduce the effect of styles. Well-known illustrations (e.g., from Disney movies) were not included in the experiment. Stimuli were presented using Psychopy 1.8.

### Electrodermal Activity

During the story, electrodermal activity was recorded using two dry electrodes attached to the forefinger and middle finger of the left hand using a GSRsensor^2^ (g-tec Gruger Technologies^©^) device. Data were acquired at 256 Hz. In one participant, register stopped after 20 min, therefore the last 3 min of story were not recorded.

### Statistical Analysis

All the analyses were performed using Matlab. Rating of the different characters before and after listening to the tale were compared using Wilcoxon signed-rank test, with two groups of images depending on their congruency in the tale (congruent: characters that presented the same moral identity in the image and in the tale; incongruent: character that presented different moral identity in the image and in the tale). Given that the ratings presented two polarities (0 for good characters; 1 for evil characters), we used two strategies to study the possible changes induced by the tale. First, we analysed the absolute value of the change between pre and post ratings. The use of the absolute value was justified to consider equivalent a change from good to evil and from evil to good, as a consequence of the tale. However, this procedure did not take into account the correctness of the change. For example, the absolute value analysis would consider as equivalent a change from 0.5 to 1 (that is, to more evil) and a change from 0.5 to 0 (that is, to more good), independently from the *correctness* of the difference, that is, whether the child correctly identified the moral identity of the character induced by the tale. Therefore, in this case, if the character was good, the variation induced by the story should be from 0.5 to 0, and the other should be considered incorrect. In order to take this into account, we recomputed the ratings for the character giving the maximum score (1) to their moral identity in the tale. This was done by inverting the scores for the good characters (1-real score) and keeping the score for the evil characters. With this procedure, all changes going in the direction of the moral identity expressed by the story would be positive, and if they went to the other direction, negative. Decision time differences between pre and post sessions were also analysed using Wilcoxon signed-rank test.

In order to analyse autonomic responses, skin conductance was low-pass filtered at 5 Hz. Electrodermal activity was time-locked to the presentation of the different pictures from 1 s before image presentation to 10 s after it. The first second before the image was set as baseline for each image. To account for individual differences on electrodermal activity, the SCR responses were standardized using the mean and variance of the activity of all images (Mas-Herrero et al., 2014). Then, standardized SCR were grouped depending on the congruency or incongruency of the images and averaged to obtain the two main conditions of study for each participant. Additionally, we also analysed the first presentation of each character and compared the congruent and incongruent conditions. Differences between conditions at each time point were assessed using Wilcoxon signed-rank test.

## Results

### Behavioural Results

In the initial test, the results confirmed that images used in the story were consistently rated as morally good or bad. On a scale from 0 to 1, with 0 being the maximum punctuation for goodness and 1 for evilness, the images proposed as representing evil characters were rated as “evil” (0.82 ± 0.22) while the images with positive moral values were rated as “good” (0.17 ± 0.19; Wilcoxon W = 2, *p* = 0.006).

**Figure 1:**
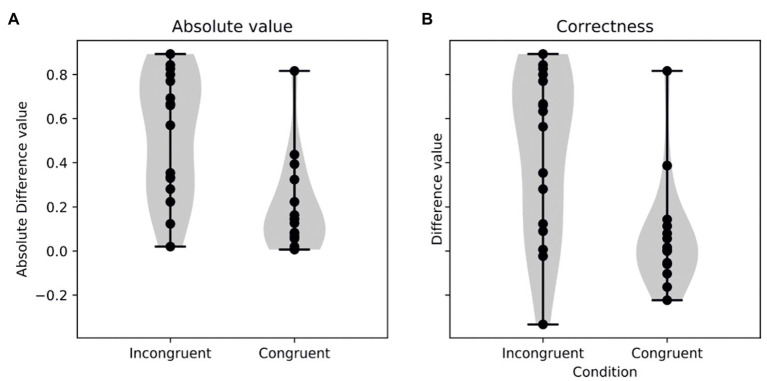
Violin plots of the absolute value **(A)** and the difference according to the correctness of the decision **(B)** of the post minus pre difference in the incongruent and congruent conditions. Note that, in the two analysis, the congruent characters presented smaller differences than the incongruent ones.

We then compared the absolute change in the rating of the images that were presented in the story, dividing them between congruent or incongruent according to their association in the tale (incongruent: good image in a bad character or vice versa; congruent: good image in a good character and bad image in a bad character). Incongruent images presented a higher change from the second session compared to the first one (absolute change in congruent: 0.19 ± 0.21; incongruent: 0.52 ± 0.28, Wilcoxon W = 14.0, *p* = 0.005; Figure 1A), demonstrating an effect in the rating of the images due to the influence of the tale.

The analysis of the correctness of the change (see Materials and Methods section) yielded similar results. Therefore, as expected, results showed a change in the correct direction in the incongruent condition (0.45 ± 0.38), but not in the congruent one (0.06 ± 0.24, W = 15.5, *p* = 0.007; Figure 1B).

Finally, there was no significant effect of the decision time before and after the tale for incongruent illustrations (5.4 ± 2.1 s before; 4.7 ± 1.0 s after, W = 42, *p* = 0.17), nor for the congruent ones (5.2 ± 1.7 s before; 4.3 ± 1.4 s after, W = 36, *p* = 0.1).

### Skin Conductance Response

**Figure 2:**
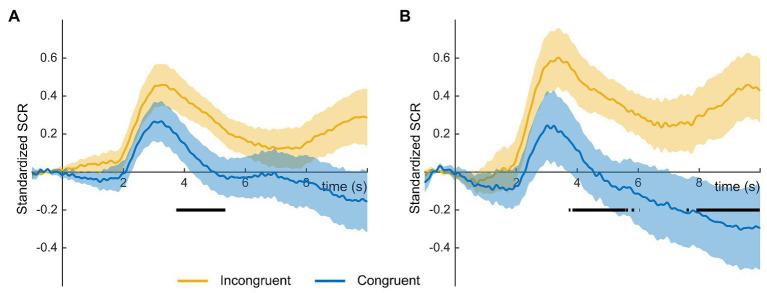
Standardized Skin Conductance Response (SCR) for the averaged **(A)** and first presentation **(B)** of incongruent (orange) and congruent (blue) illustrations during the story. Shaded areas indicate the SEM for each condition, and solid black line indicates the time points where the difference between condition was significant (*p* < 0.05).

Electrodermal activity locked to the image presentation revealed an increase for both incompatible and compatible images, in both the averaged (Figure 2A) and the first presentation (Figure 2B) of the illustrations. Incompatible images presented a higher increase compared to compatible ones. Point by point Wilcoxon test revealed significant differences among the activities for the two conditions in a time range from 3.7 to 5.3 s after image onset in the averaged images (Figure 2A), and from 3.8 to 5.6 s and from 7.9 to 10 s after image onset only taking into account the first presentation of the images (Figure 2B).

## Discussion

In the current paper, we present a new experimental paradigm to study the perception of moral identity and its change through experimental manipulation in children. We used the combination of an audio story and the presentation of pictures of characters with different features (which induce *a priori* associations with either good or evil moral identities) to study the changes in the rating of the images of such characters and their physiological responses (specifically, SCR) associated with incompatibility between characters’ perceived moral identities and their behaviour. Results showed that incompatibility between *a priori* moral identity (defined by the physical characteristics of the image) and the behaviour of the character induced an increased SCR.

The first important result of the paper is that the presentation of images with visual content that was incongruent with the actions of the represented character produced a significant increase in SCR compared with congruent images. Interestingly, the SCR pattern was compatible with very fast response (peak at 3 s, in the minimum range of SCR response), supporting the idea that children very quickly realized the incongruence between the image and the story. These results suggest two important aspects: first, that SCR is a sensitive measure of moral incongruence; and second, that children between 8 and 10 years old can detect such incongruences and respond very quickly to them. Therefore, the present results support the existence of a link between perceived physical traits and moral traits “…unattractiveness is bad” and “beauty is good” (Dion et al., 1972; Griffin and Langlois, 2006; Lemay et al., 2010; Ma et al., 2016). However, it is important to note that while children easily identified the moral identity of the images (both at the first test and then while listening to the tale), the present experiment does not identify which specific visual elements are used by children in determining moral identity from illustrations. Therefore, different characteristics, such as weight, beauty, or even facial expressions (e.g., an angry face might be associated with evil characters) could influence the decision of the participants. For example, an untrustworthy face might present several features – such as a downturned mouth with thin lips, an angry expression (Caulfield et al., 2016), or angry-looking eyes – that coincide with the evil profile of the sample of characters of our research. In contrast, illustrations of trustworthy faces might present other characteristics, such as smooth skin or small face sizes and, in some cases, smiling mouths (Dotsch and Todorov, 2012) and/or small brow ridges, chins and noses (Ma et al., 2016), or happy expressions (Caulfield et al., 2016), and be seen in female faces more than male faces (Dong et al., 2018). Although it is well-known that some features such as eyebrows (Tipples et al., 2002) and expressions are crucial in deciding the moral identity of a character, our experimental manipulation does not provide knowledge of what the critical features were in the decision that allowed categorization of a moral model. Future studies could incorporate this element by directly asking the children or by using other techniques such as eye tracking, to determine which elements of an image capture the attention during decision-making (Dotsch and Todorov, 2012; Ma et al., 2016; An et al., 2017).

The second main finding of the present study is that children changed the moral identity assigned to an image when faced with behaviours that contradicted it. Therefore, after listening to the story, children were able to switch the ratings of the characters showing incongruence between their induced moral identity and their actions in the tale, ignoring the image content and focusing on their behaviour. This is an important result, as it suggests that children might change their intuitive decisions about an image when confronted with the facts (Rahman, 2011). Nevertheless, the judgement of being trustworthy or not seems affected by context (Dong et al., 2018). At the same time, this change is puzzling in terms of the interpretation of the data on the basis of current theories on moral decision. SCR of congruent and incongruent images presented significant differences at approximate 4 s, which is a fast response considering the time resolution of the technique. This would support the idea that this incongruency effect was very automatic, a kind of “affective valence” response following the idea of Greene and Haidt (2002). Indeed, our results suggest that the moral incongruency triggered an automatic and fast response (Haidt, 2001), probably due to its emotional content (Greene and Haidt, 2002). However, when appearances were deceptive (that is, when children had real information about the characters and their circumstances that contradicted the first impression), the children were able to change their criterion, yielding to a conscious decision in contrast to the first fast and intuitive one. Therefore, this decision was no longer automatic, but made with knowledge of cause, supporting a rationalistic view.

The study also presents some limitations. First, only one story was used in the experiment. Although we consider that the story was appropriate, as it presented different characters doing different actions (morally good or bad) and was not very well-known, which allowed the attention of children to be held, we cannot know whether the effects found can be generalized to other stories. Future studies could try to replicate these effects using other tales with different characters and plots. In addition, the congruency effect was not counterbalanced; that is, the characters always presented the same congruency or incongruency between the illustration and their moral identity. Although both the behavioural and psychophysiological results are consistent, an interesting manipulation would be to counterbalance the congruency of the story characters (e.g., the illustration of one character was congruent with his/her behaviour in half of the participants and incongruent in the other half) to minimize possible effects of one particular character, illustration, or combination of both.

In conclusion, present results show that it is possible to measure the reaction of children in the face of moral congruencies driven by the features of a character. This might open up new ways to study the visual codes associated with judgements on the basis of physical features. Although the focus of the study was the decision about the moral identity of characters (good vs. evil), future studies could be devoted to assess other topics such the relationship between physical appearance and identity (Griffin and Langlois, 2006), the physical appearance of “evil” animals (Lee and Kang, 2012), and the impact of certain physical elements, such as eyebrow position (Tipples et al., 2002; Ma et al., 2016), social features from faces (Dotsch and Todorov, 2012), or gender (Dong et al., 2018).

## Data Availability Statement

The raw data supporting the conclusions of this article will be made available by the authors, without undue reservation.

## Ethics Statement

The study was reviewed and approved by the University of Barcelona’s Bioethics Commission. Written informed consent to participate in this study was provided by the participants’ parent or legal guardian.

## Author Contributions

NO-S and JM-P designed the experiment, performed the experimental sessions, and wrote the manuscript. JM-P analyzed the data. All authors contributed to the article and approved the submitted version.

### Conflict of Interest

The authors declare that the research was conducted in the absence of any commercial or financial relationships that could be construed as a potential conflict of interest.
